# An open-sourced, web-based application to improve our ability to understand hunter and angler purchasing behavior from license data

**DOI:** 10.1371/journal.pone.0226397

**Published:** 2020-10-01

**Authors:** Nathaniel B. Price, Christopher J. Chizinski, Joseph J. Fontaine, Kevin L. Pope, Micaela Rahe, Jeff Rawlinson

**Affiliations:** 1 School of Natural Resources, University of Nebraska, Lincoln, Nebraska, United States of America; 2 Nebraska Cooperative Fish and Wildlife Research Unit, and School of Natural Resources, University of Nebraska, Lincoln, Nebraska, United States of America; 3 U.S. Geological Survey—Nebraska Cooperative Fish and Wildlife Research Unit, and School of Natural Resources, University of Nebraska, Lincoln, Nebraska, United States of America; 4 National Wild Turkey Federation, Lincoln, Nebraska, United States of America; 5 Nebraska Game and Parks Commission, Lincoln, Nebraska, United States of America; Shandong University of Science and Technology, CHINA

## Abstract

State fish and wildlife agencies rely on hunters and anglers (i.e., sportspersons) to fund management actions through revenue generated from license sales and excise taxes on hunting and fishing equipment. There is a need to develop new techniques that bridge the information gap on participation and provide agencies with an understanding of sportspersons at a resolution that can more directly inform efforts to engage sportspersons. Monitoring sportsperson participation using information about their license-purchasing behavior has the potential to reveal important patterns in recruitment (first-time purchase of a hunting or fishing license), retention (continued purchase of licenses across multiple years), and reactivation (purchase a license after several years with no purchases). Providing up-to-date information on what licenses are purchased, when and by whom may prove invaluable to managers and policy makers. We present a customizable, open-source, web-based application—huntfishapp—that allows the user to query and interact with a structured query language (SQL) hunting and fishing license database. The huntfishapp serves as an informational resource and tool that provides a framework to share information on license sales across an agency, with intent of increasing understanding of (a) sportspersons and (b) how management decisions affect sportspersons. Data dashboards, like the huntfishapp, allow agencies and non-governmental organizations to become more knowledgeable of their customer base and provide a greater understanding of management-decision effects on hunting and fishing participation.

## Introduction

State fish and wildlife agencies rely on hunters and anglers (i.e., sportspersons) to fund management actions through revenue generated from license sales and excise taxes on hunting and fishing equipment. The continued decline in hunting and fishing [1–4], therefore, constitutes a threat to conservation and management in North America [5, 6]. Non-governmental organizations and state agencies concerned over the loss of sportspersons are increasingly investing in programs focused on improving recruitment, retention, and reactivation of sportspersons (hereafter R3 programs); however, the success of R3 programs remains largely unknown in part because agencies lack a detailed understanding of patterns and processes that drive sportsperson participation.

Conducted every 5 years, the National Survey of Fishing, Hunting, and Wildlife-Associated Recreation (e.g., [1]) identifies general patterns of sportsperson participation in the United States of America. Although useful for understanding overarching trends, the spatial and temporal scales, as well as the limited sampling regime of the National Survey, provide little value for understanding participation within a given state, among demographic groups, or between agency fiscal years—information that is key to building a successful fish and wildlife management strategy. To increase the resolution of their understanding of sportsperson participation, agencies conduct additional surveys (e.g., [7, 8]). Unfortunately, due to the high costs of interviewing people, state surveys are often conducted sporadically and with specific aims, which can limit their application to specific locations, user groups, and years. Given the challenges of traditional approaches, there is a need to develop new techniques that bridge the information gap on participation and provide agencies with an understanding of sportspersons at a resolution that can more directly inform R3 efforts.

Monitoring sportsperson participation using information about their license-purchasing behavior has the potential to reveal important patterns in recruitment (first-time purchase of a hunting or fishing license), retention (continued purchase of licenses across multiple years), and reactivation (purchase a license after several years with no purchases) [9]. Providing up-to-date information on when and what licenses are being purchased and by whom can prove invaluable to managers and policy makers directing R3 programs and informing public marketing and communication strategies [10, 11]. Despite the apparent value of license-purchase data to understanding sportsperson participation, few agencies have a strong grasp of how purchasing patterns fit into R3 objectives. Developing, maintaining, and analyzing databases that can provide such information requires data-science skills, which effectively make the important R3 information that is available from license-purchase data unattainable for most personnel in fish and wildlife agencies. Recent advances in computer software have made it possible to overcome traditional data management and analysis challenges by creating computer applications, or dashboards, that are more user friendly and thus more broadly available to agency personnel [12]. Unfortunately, to date there are few customer applications that have been specifically designed for fish and wildlife agencies, and those that exist can be cost prohibitive and generally lack the flexibility necessary to meet the diverse needs of multiple agencies.

Herein we present a customizable, open-source, web-based application that allows the user to query and interact with a structured query language (SQL) database containing repeat-purchase data on the sale of licenses (e.g., fishing), permits (e.g., deer), passes (e.g., park), certificates (e.g., hunter education), and other related items. We present the web-based application with its full source code and a sample dataset of repeat-purchase data for 10,000 customers purchasing fish, deer, hunt, hunt-fish combo, spring turkey, and fall turkey licenses and permits in Nebraska between 2008 and 2018. The application is designed to be easily used with databases at other state agencies. Using the web-based application with a different database backend only requires the database administrator to create a view with the necessary, non-personally identifiable data in a standard format and to modify a configuration file with some state-specific information. To minimize the amount of effort required to adapt the application to other states, the application also includes county-level maps and population estimates by county, sex, and age group for each state in the continental United States of America. The goals of creating the open-source, web-based application are to (1) facilitate other states’ ability to make data-driven decisions that will, in turn, benefit all states (e.g., through non-resident sales), (2) facilitate the mutually beneficial sharing of information between states in the form of directly comparable visualizations and data summaries, and (3) encourage others to contribute to the continued development of an open-sourced, web-based application rather than duplicating efforts in each state or seeking proprietary, commercial solutions.

## Methods

### Application development

The web-based application (hereafter “app”) was developed in direct partnership with teams at Nebraska Game and Parks Commission (NGPC). Members of the R3 group at NPGC had a list of information that would be most useful to them to better understand sportsperson dynamics. Further, the R3 group had knowledge of some dashboards and generated feature requests based on dashboards from states such as Iowa, Oregon, and Arizona. The team at the University of Nebraska-Lincoln built the basic structure of the app, as described below, and met regularly with the R3 group to make improvements to the app. After a mature version of the app was developed, we met with larger groups of NGPC such as the Fisheries Division, Wildlife Division, and Communications Division to get further insight into potential design comments and applications of the app. The process to design the app with stakeholder input required meeting the needs of specialized users (e.g., desiring up-to-date participation numbers on a daily basis) and less-specialized users (e.g., interested in general trends among customers). The iterative approach that we took in designing the app helped us achieve a balance in the app utility between these groups.

### Ethics statement

All research protocols were reviewed prior to the start of this research project. All research protocols and techniques were approved by the University of Nebraska–Lincoln Institutional Review Board (IRB Approval #: 20160616155 EP) and complied with NGPC policies. The need for consent was waived by the ethics committee.

### Application structure

**Fig 1:**
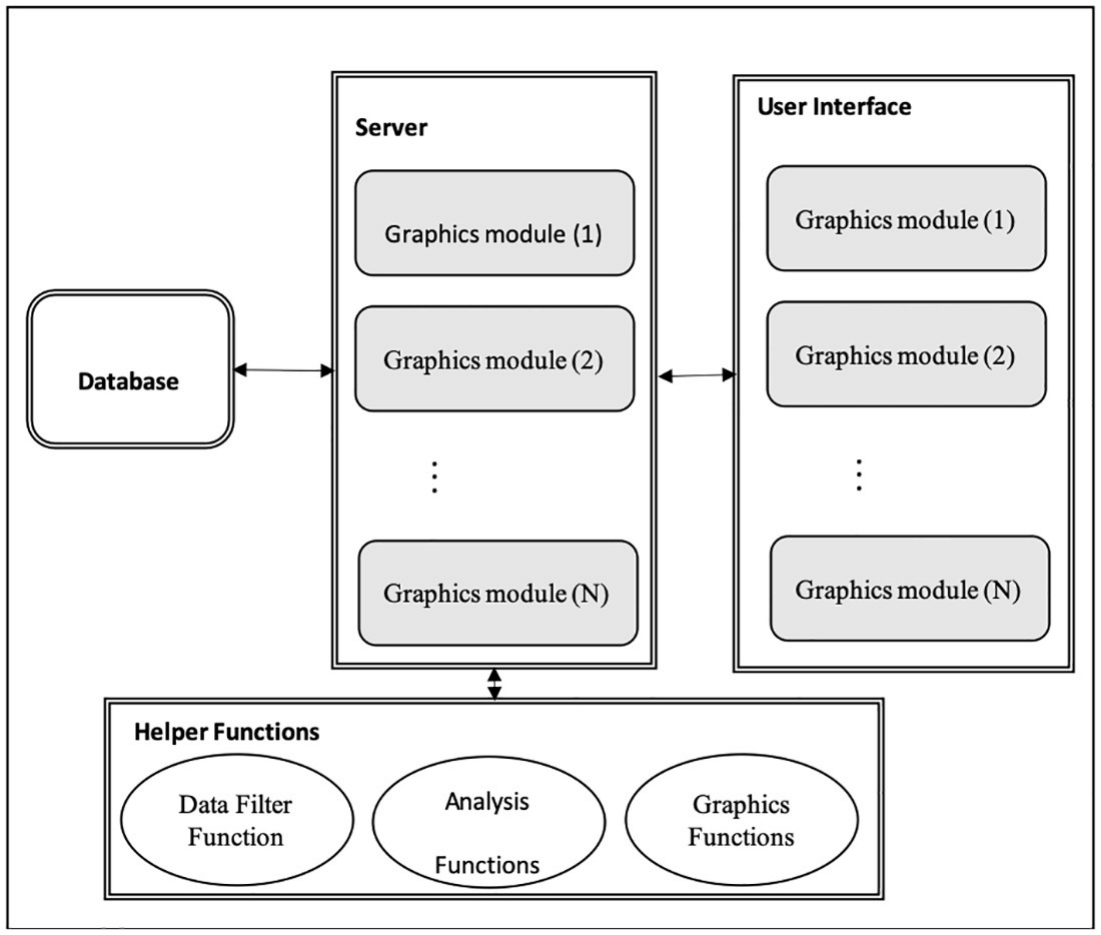
Diagram illustrating the organization of the app. The app uses a modular design with separate server and user interface (UI) functions for each interactive graphics module. Graphics modules call non-reactive helper functions to filter data, analyze data, and generate graphics. Separate modules are used to handle downloading plots and data summaries (not shown).

The app and supporting functions are distributed as an open-source package called “huntfishapp” written in the programming language R [13]. The app was created using the package “shiny” [14]. Shiny is an open-source R package that provides a powerful web framework for building web-based applications using R. The app utilizes a modular design. Shiny modules allow a large Shiny application to be separated into isolated modules that each have their own server functions and user interface (UI) [15, 16]. In the app, there are nine modules for interactive graphics, one module for downloading plots, an------d one module for downloading data summaries (Fig 1). The graphics modules allow the app to be easily extended to handle new data-analysis tasks through additional modules and facilitate easier reasoning and debugging by isolating tasks. The modular design allows for a flexibility that works well with an agile software development approach (see [17].) The modules for downloading plots and data summaries are called by each graphics module. These modules are used to avoid duplication of server-side logic and UI that is used repeatedly. An additional module is used to separate the initial login screen from the primary UI.

#### Graphics modules

Each graphics module shares a similar structure. The UI functions for each module use a tabular design with tabs for the graphic, graphic description, graphic options, formatting and downloading the graphic, and viewing and downloading graphic data as a comma-separated-values (CSV) file. The UI functions are easily extended by adding additional tabs as existing requirements change or new requirements arise. The server functions for each module also share a similar structure. The server functions apply data filters, define grouping variables based on user input, summarize data by applying a specific analysis function, create and render a graphic, and provide tooltip and click actions for graphics when applicable. The graphics modules call separate modules that contain server functions and UI for downloading graphics and analysis results. Analyzing the data and generating plots is performed by a set of analysis and plotting functions.

#### Analysis functions

The analysis functions use the “dplyr” [18] and “dbplyr” [19] packages. The aim of the “dplyr” package is to provide a function for each basic verb of data manipulation. The “dbplyr” package provides a backend for databases so that queries coded in R can be translated into database queries. The majority of the required computation is handled by the database backend, rather than the server hosting the app, because the analysis functions send queries to the database that return only summary results. The use of “dplyr” and “dbplyr” is convenient for programming, and also serves as a middle man between the user and the database to sanitize user-designed queries and prevent SQL injection attacks [20]. Furthermore, the app is not restricted to a specific database language because the necessary SQL translation is performed by the “dbplyr” package. The analysis functions facilitate testing each analysis component because they are isolated (i.e., unit testing) and non-reactive (i.e., they do not automatically execute when dependencies change). Separating the reactive components from the non-reactive components makes it easier to isolate errors that arise due to the reactive (i.e., dynamic) nature of the app.

#### Graphic functions

The graphic functions primarily use the “ggplot2” package [21]. Although there are other packages that are designed specifically for interactive graphics [22], the “ggplot2” package was selected for the ability to reliably create professional-quality graphics that are easily downloaded. In particular, the “ggplot2” package excelled at creating subplots (i.e., facet plots) conditional on a particular variable (e.g., residency). The graphics functions are separated by the type of graphic created (e.g., bar plot, line plot, choropleth map). The graphic functions ensure consistency in graphics across modules, avoid repetition of code, and facilitate unit testing.

#### Database backend

Before analysis functions can be applied, data must be gathered from multiple database tables into a form that can be easily analyzed. Database structure and available data will vary between state databases; thus, the app interacts with the database backend through a SQL view, named “huntfishapp,” containing the required, non-personally identifiable data in a standard format. The app requires 14 variables (Table 1). The SQL view is a stored query that acts like a virtual table and will be specific to the database structure. This view should be constructed by the database administrator to allow the app to interact with the SQL database. The database containing the view should be added as an ODBC (Open Database Connectivity) data source on the machine running the app. The app will attempt to establish a connection to the database by looking for a data source under the default name “license_data.” On startup, the user will provide a username and password to connect to the database and these credentials are verified by attempting to perform a “select” operation on the “huntfishapp” view.

**Table 1:** Description of required variables and formatting for the "huntfishapp" view.

Variable	Description
itemUID	Integer—Unique identifier for each item (permit, license, stamp, etc.).
customerUID	Integer—Unique identifier for each customer (owner of the item)
issueDate	Date (YYYY-MM-DD)–Date item was issued or sold
itemResidency	Character—Residency designation for item with “T” for resident and “F” for non-resident
duration	Character—Description of duration attribute for each item (e.g., “Lifetime”)
durationValue	Numeric—Duration of item coded as numeric value
residency	Character—Residency attribute for customer with “T” for resident and “F” for non-resident
state	Character—State of residence attribute for customer as two-letter abbreviation (e.g.,”NE”)
county	Character—County of residence attribute for customer in lowercase
gender	Character—Gender attribute for customer with “Female,” “Male,” and NULL values
age	Numeric—Age attribute for customer at time of purchase
itemType	Character—Description of item type (e.g., “Deer”)
itemYear	Numeric—Year attribute for item. May or may not correspond to year of purchase
price	Numeric—Price attribute for item including applicable fees

The app uses a function to build an internal data view (i.e., stored SQL query), from the “huntfishapp” view on the server, conditional on the active data filters. This function is constructed from “dplyr” data manipulation verbs wrapped in simple functions that accept an additional logical argument. For example, the wrappers for “filter” accept a logical argument (e.g., is filter active) and return the manipulated data frame when the argument is true or the original data frame when the argument is false. More precisely, these functions return a “dplyr” data frame object that stores the query construction until the query is executed at a later point. The functions used to create these data views provide a simple means of combining global data filters (i.e., filters active in all graphics modules) with local data filters (i.e., filters specific to a graphics module).

The app also provides an option at startup to load data from a CSV file rather than connecting to a database. If this option is selected, the app will run using internal sample data. This functionality is provided for demonstration and testing purposes. Although it is very quick to perform analyses when the full dataset is loaded into memory, it may not scale well to large datasets and multiple simultaneous users. Therefore, it is recommended that the app be used with the SQL database backend.

### User interface

The UI for the app is comprised of a side panel on the left with data filters and collapsible panels that span the remaining page width on the right. There is a single panel for each graphics module. The app has a minimalist design where only one panel is permitted to be expanded at any time. Having only a single active (i.e., expanded) panel prevents the app from running potentially time-consuming SQL queries to update plots that are not of interest to the user. When a panel is collapsed, the reactive code responsible for executing SQL queries is paused. When the user activates a panel (i.e., expands it) then the necessary SQL query is immediately executed. The SQL query will automatically re-execute whenever the panel is active *and* results are invalidated (e.g., user inputs change). Therefore, if the user switches to another panel and returns to the previous panel, then the query will not re-execute because the dependencies did not change. Furthermore, if the user changes the global data filters on the separate global filters side panel, then invalidated graphics will not re-execute until the respective panels are activated. Therefore, the collapsible panel design serves to minimize the load on the server, increase responsiveness of the app, and avoid distracting or overwhelming the user with the simultaneous presentation of complex graphics.

An important component of the app design is communicating to the user when the application is busy. This is particularly important because the open-source version of Shiny server does not allow for parallel processing. The R program hosting the app runs on a single processor and if this processor is busy with a SQL query, then the app will be temporarily unresponsive. Moreover, when multiple users are accessing the app, a time-consuming query from one user will cause the app to become unresponsive for other users because tasks are completed sequentially. To improve the responsiveness of the app, we used the “promises” [23] and “future” [24] packages to spawn additional processes to allow SQL queries from different users to be sent to the database in parallel. To communicate the progress of queries to the user, we show a small notification indicating that a query has been sent and identifying the graphics module that initiated the query. The notification remains until the query completes and then a second notification briefly appears to indicate that the query was completed along with the time it took to complete the query. Displaying the wait times improves the user experience because the wait times are objectively short (typically 5 to 10 seconds), but might be perceived as long if no messages were displayed.

#### Filter side panel

**Fig 2:**
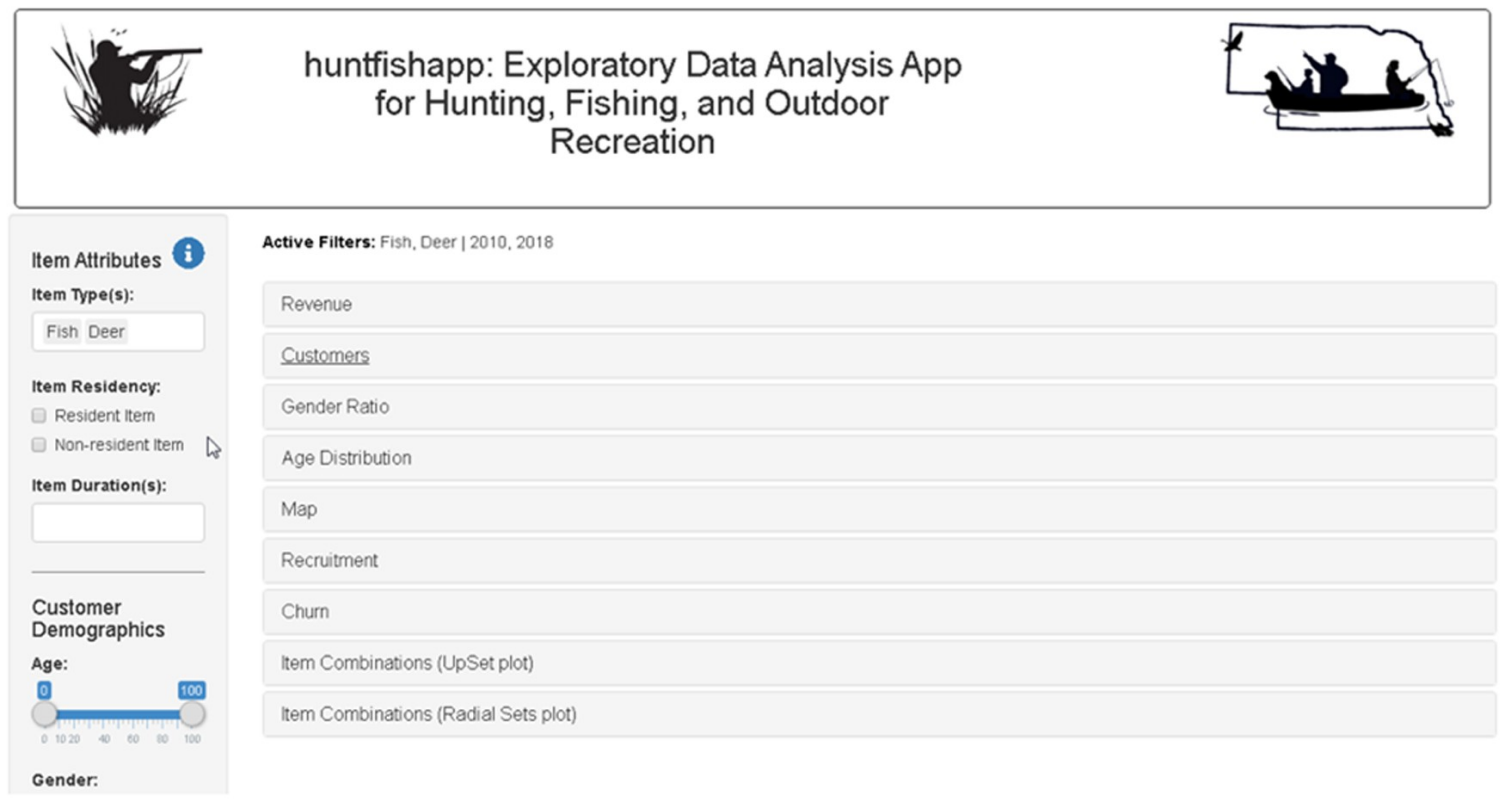
The app layout consists of a sidebar with data filters on the left and collapsible panels on the right.

The filter side panel provides global filters that affect all graphic modules. It is easy to select a particular segment of the customer population because the filters are global, and then explore the various graphics modules for the selected customer segment. The filters are broadly grouped into item attributes, customer demographics, purchase date, and customer address (Fig 2).

#### Revenue panel

**Fig 3:**
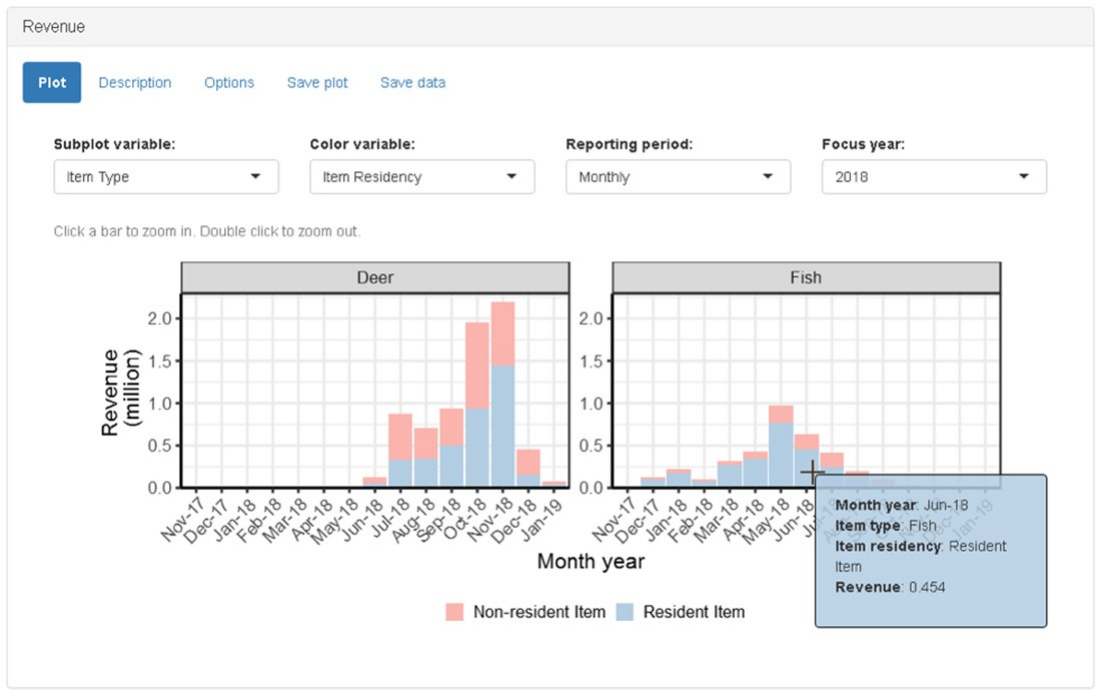
Screenshot of revenue panel when “item type” is selected as subplot variable, “item residency” is selected as color variable, and “monthly” is selected as reporting period. For simplicity, only two item types are shown; additional rows are added automatically to accommodate any number of subplots. The plot includes permits issued in 2016 prior to the start of the 2017 permit year and late season permits issued in 2018.

The revenue panel calculates the sum of the price variable and displays a bar graph of revenue according to the user-specified reporting period (Fig 3). The default reporting period is “annually” and the bar graph displays total revenue by year. When the reporting period is “annually,” the plot also includes a horizontal line indicating the average revenue for the previous five years. Revenue is automatically reported in units of millions or thousands to simplify the y-axis labels. The panel is designed to allow the user to “zoom in” or “zoom out” by *simultaneously* changing reporting period (i.e., annually, monthly, daily) and filtering data (i.e., focus year, focus month). The primary method of triggering this action is to click on a particular bar in the graph to zoom in and double-click anywhere on the plot to zoom out. For example, if the user clicks on the bar corresponding to revenue for year 2015, then the reporting period is set to monthly and the focus year is set to 2015, and the resulting bar graph displays total revenue by month during 2015. If the user then clicks on the bar corresponding to revenue in May of 2015, then the reporting period is set to daily and the focus month is set to May, and the resulting bar graph displays total revenue by day during May 2015. A double click action anywhere on the plot will decrease the temporal resolution by one-step (i.e., daily to monthly; monthly to yearly). Help text is displayed above the graphic to explain the action of clicks and double-clicks. The “reporting period,” “focus year,” and “focus month” inputs can also be controlled using drop-down menus above the graphic. These inputs serve to provide informative labels when using the click actions. The “focus year” and “focus month” inputs are hidden when not applicable (e.g., focus month is hidden when reporting period is annually).

Two additional drop-down menu inputs above the graphic allow the user to specify grouping variables in the revenue calculation and assign these variables to graphic aesthetics. The “subplot variable” input allows the user to split the bar graph into multiple subplots (facets in “ggplot2” terminology) conditional on the value of a particular variable. For example, selecting “residency” from the “subplot variable” drop-down will result in a grid of three bar graphs displaying total revenue by reporting period for “non-resident,” “resident,” and “(missing residency information)” sportspersons. An additional radio-button input on the “options” tab of the panel specifies whether the y-axis scales for the subplots are the “same scales” or “different scales.” Choices for “subplot variable” are “total” (i.e., single bar graph), “item type,” “gender,” “residency,” “item residency,” “duration,” and “age group.” The second drop-down menu input allows the user to select a “color variable.” The “color variable” is used to assign a variable for creating a stacked bar graph (i.e., bar heights do not change but they are colored in proportion to relative contributions). The choices for the “color variable” are the same as for the “subplot variable.”

In addition to the click interaction described above, the revenue panel graphic also has tooltip interactions. Hovering the mouse pointer over a bar will display the reporting-period value and the corresponding revenue value in a tooltip display. The tooltip will also display the values for the subplot and color variables, when applicable. If using a color variable, then the tooltip value corresponds to a particular section of the stacked bar and the tooltip color matches the color of the relevant section.

#### Customers panel

The customers panel calculates the total number of individuals, or alternatively the total number of items, and generates a bar graph of the count according to the user-specified reporting period. The subplot variable, reporting period, click actions, and tooltip are similar to the functionality described for the Revenue Panel. The customers panel also has a “primary variable” drop-down menu that allows the user to switch between a count of customers and a count of items. If a count of items is displayed, then a “color variable” input is available with the same functionality as described for the Revenue Panel. The color variable is not available when the bar graph displays a count of customers because individuals may belong to multiple groups such that the total number of customers each year (i.e., total bar height) is not the same as the sum of the groups (i.e., stacked bar height).

#### Gender ratio panel

**Fig 4:**
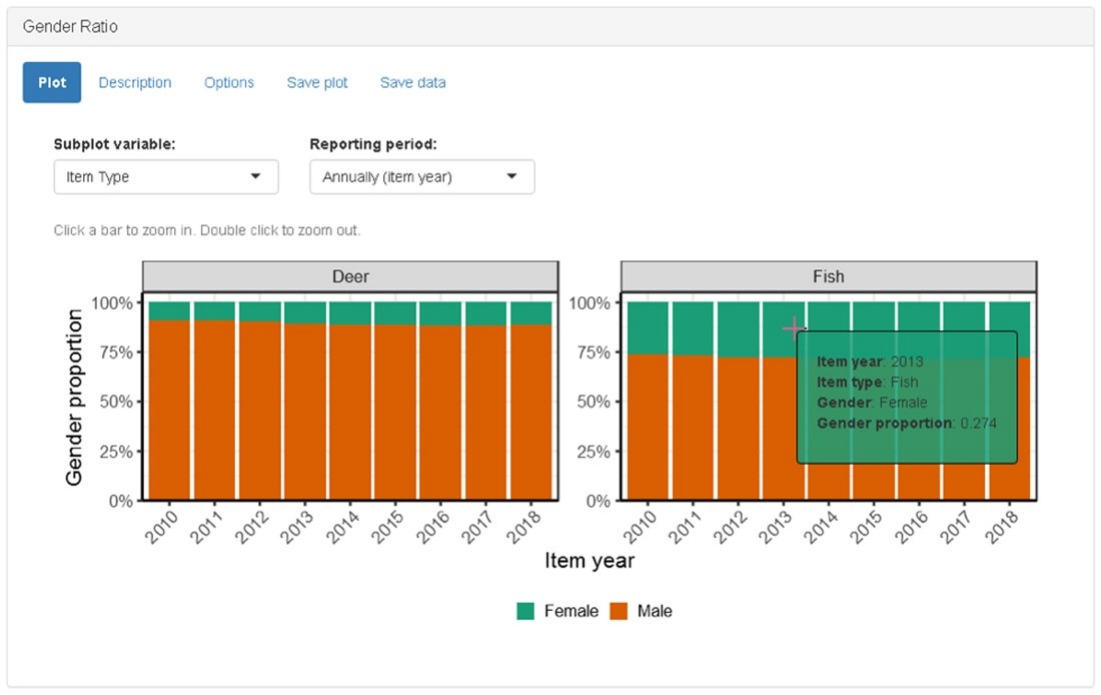
Screenshot of gender ratio panel when “item type” is selected as subplot variable. For simplicity, only two item types are shown; additional rows are added automatically to accommodate any number of subplots.

The gender ratio panel calculates the proportion of male and female customers and generates a 100% stacked bar graph according to the user-specified reporting period (Fig 4). The subplot variable, reporting period, click actions and tooltip are similar to the functionality described in the Revenue Panel.

#### Age distribution panel

**Fig 5:**
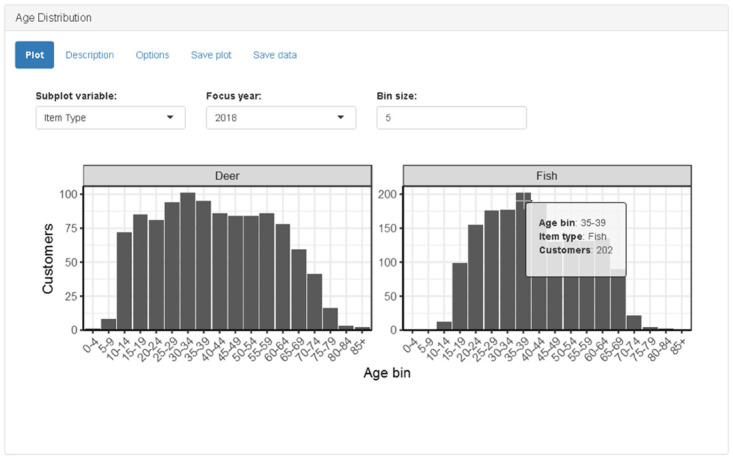
Screenshot of age distribution panel when “item type” is selected as subplot variable. For simplicity, only two item types are shown; additional rows are added automatically to accommodate any number of subplots.

The age distribution panel calculates and displays a histogram of the number of customers according to the user-specified bin size (Fig 5). The bin size is controlled by a numeric “bin size” input. The subplot variable and tooltip are similar to the functionality described in the Revenue Panel. The graphic has no click actions. The age distribution is only calculated for a single year. The “focus year” drop-down menu input allows the user to select the year.

#### Map panel

**Fig 6:**
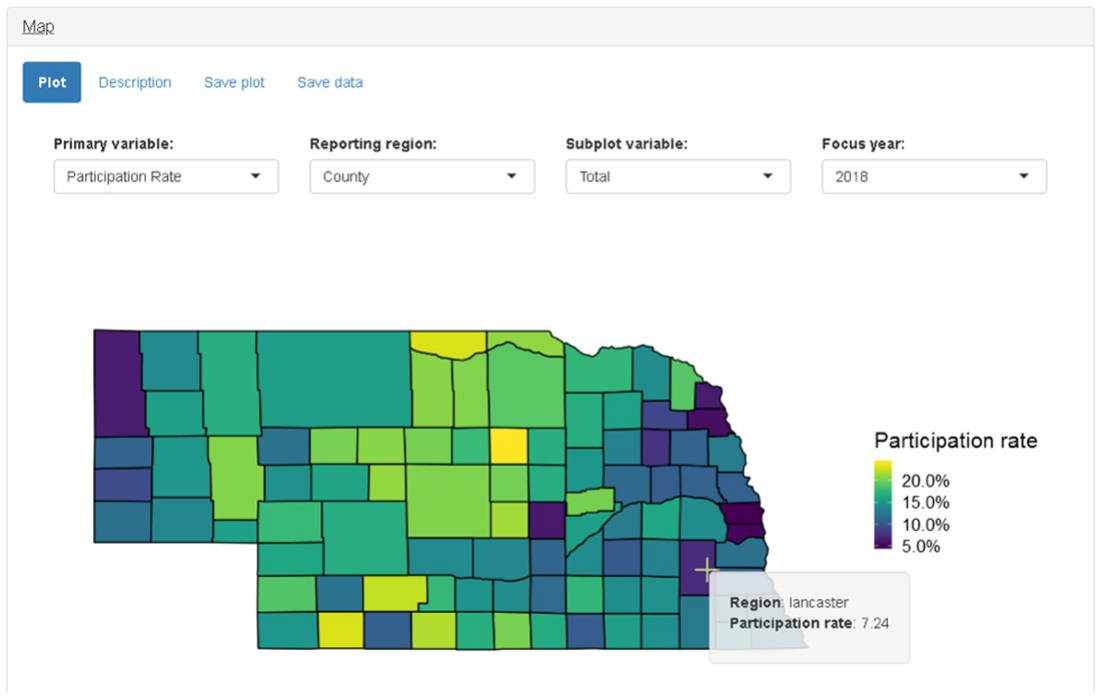
Screenshot of map panel when “participation rate” is selected as primary variable and “county” is selected as reporting region.

The map panel displays choropleth maps by county or state for a given year. A “reporting region” drop-down input allows the user to select a county or state map (Fig 6). The map panel can display the number of items, number of customers, churn rate (percent of customers who do not purchase in next 5 years), recruitment rate (percent of customers who did not purchase in last 5 years), and participation rate (percent of population who purchased based on American Community Survey 5-year population estimates from U.S. Census Bureau). The “primary variable” drop-down input allows the user to select from these different choices. All values displayed in the map are for a single year that is controlled by the “focus year” drop-down menu input. The “subplot variable” input allows the user to create multiple maps for different groups that all share a common scale.

#### Churn panel

The churn panel displays information regarding trends in customer churn (i.e., customer attrition; permanent loss of customers). It can be challenging to determine how many customers are lost each year because customers may have gaps of one or more years between purchases. The app performs a moving window analysis that looks at recent purchase records to determine which customers were lost in prior years. The churn calculation does not account for the duration variable (e.g., multiyear, lifetime). By default, customers are considered lost if they do not re-purchase within 5 years. The choice of a window size is subjective and there is a tradeoff between accuracy and obtaining up-to-date results. For example, results from an analysis using a 5-year window may not reflect current trends because the most recent churn estimate available during 2018 is for 2013 purchases. If the window size is too small, then churn will be overestimated because customers with relatively large gaps between purchases will be incorrectly classified as permanently lost. If the window size is sufficiently large, then increasing the window size will not significantly change the estimate of the number of lost customers. For example, the number of customers who did not re-purchase within 5 years is approximately equal to the number of customers who did not re-purchase within 6 years because the majority of these customers will never purchase again (i.e., they are permanently lost). The preferred window size may vary based on customer demographics if some groups exhibit large gaps between purchases relative to other groups. A slider input, available on the “options” tab, allows the user to override the default setting and control the size of the moving window.

**Fig 7:**
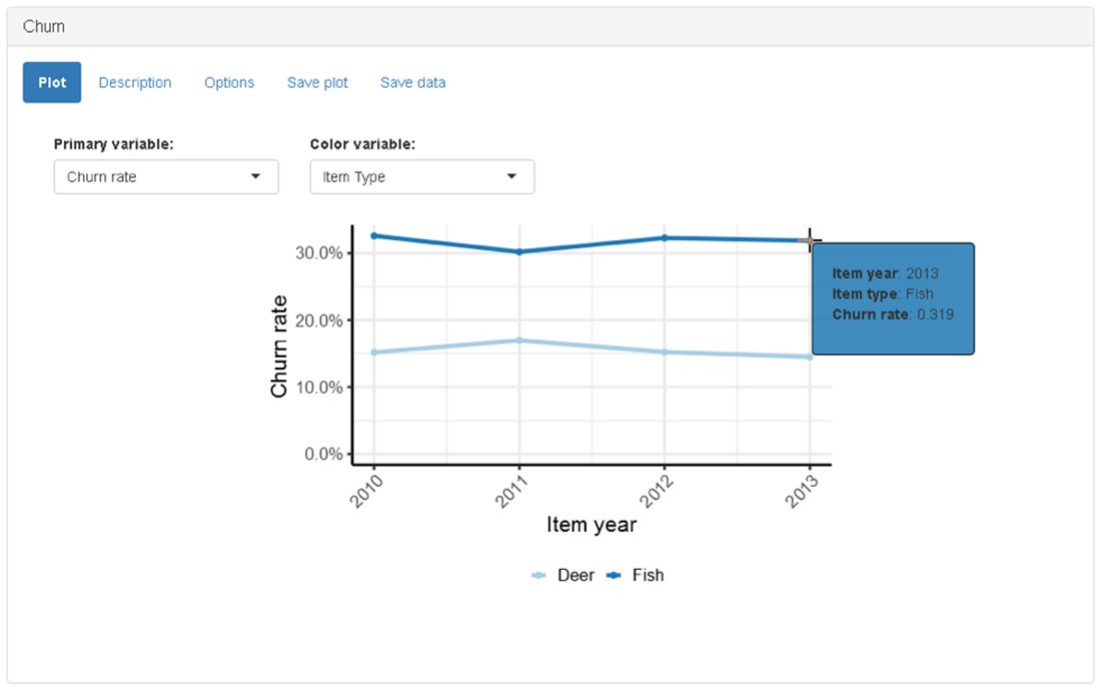
Screenshot of churn panel when “item type” is selected as color variable.

The churn panel has a “primary variable” drop-down menu input that allows the user to select churn rate, retention rate, number of churned customers, or number of retained customers (Fig 7). Retained customers are considered to be the complement of churned customers (i.e., customers that don’t churn are retained). The churn and retention rates are the proportions of churned and retained customers, respectively, relative to the total number of customers who purchased a license in a given year; churn and retention rates for a given year add to 100.

#### Recruitment panel

The recruitment panel displays information regarding trends in customer recruitment. It is challenging at the start of an electronic license database system to distinguish between new customers and returning customers. The recruitment panel displays a graphic similar to the graphic described in the Churn Panel. The analysis is also based on a moving window. However, instead of looking at recent purchases to determine which customers are lost, the recruitment analysis looks at historical purchases to determine which customers are new. By default, customers are considered new recruits if they did not purchase in the previous five years. The recruitment panel has a “primary variable” drop-down menu that allows the user to select between number of customers or recruitment rate. The recruitment rate is defined as the number of new customers divided by the total number of customers that purchased a license in the previous year. The recruitment rate is defined with respect to the previous year so that it can be compared to the churn rate. In particular, if the churn rate is approximately equal to the recruitment rate, then the number of active customers is relatively constant across years.

#### Item combinations (UpSet plot) panel

**Fig 8:**
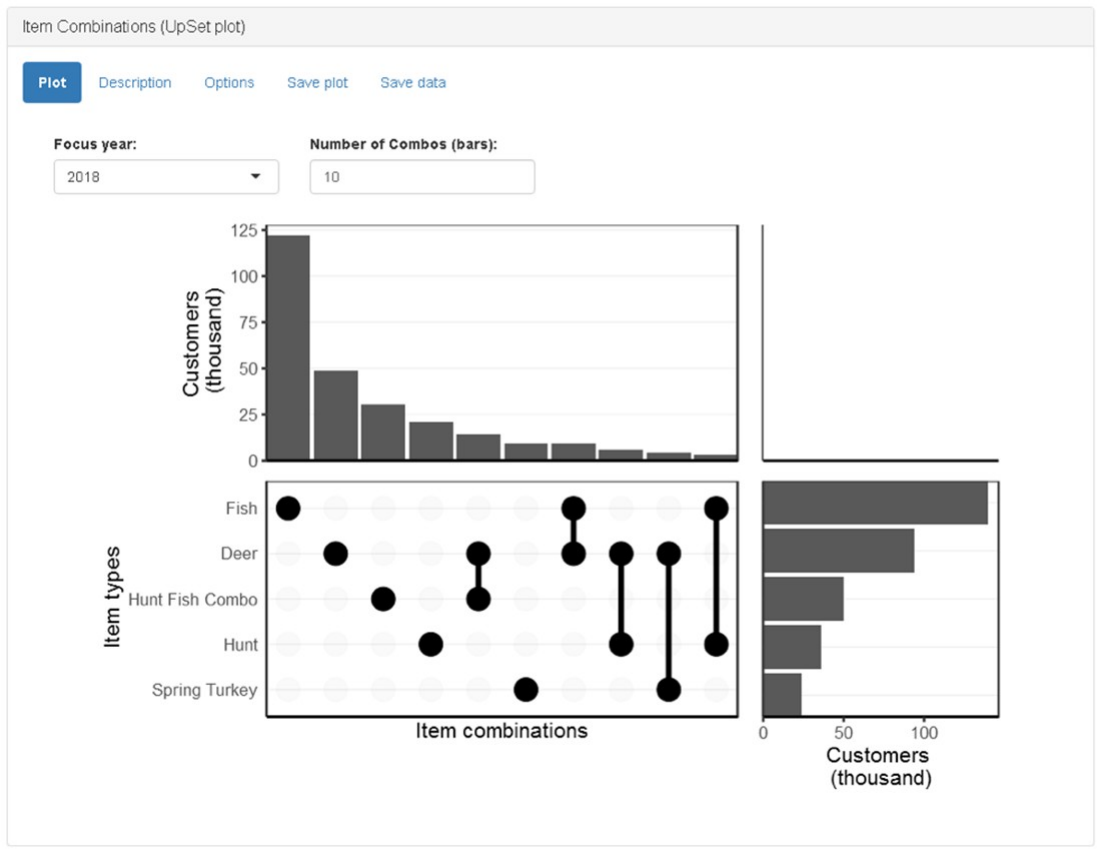
Screenshot of item combinations (UpSet plot) panel.

Customers may purchase a variety of items (e.g., fishing license, deer permit, waterfowl stamp, etc.). The groups of customers purchasing different items form overlapping sets. For example, customers may purchase only a fishing license, only a deer permit, or both fishing license and deer permit. Therefore, we have a “deer” set and a “fish” set, and we may be interested in the intersection of these two sets. A Venn diagram is useful for visualizing overlapping sets when there are few groups. However, it is challenging to interpret a Venn diagram that accurately represents all the overlapping sets when there are many groups. The permit combination panel displays a graphic based on an UpSet diagram (Fig 8) [25]. In our simplified version of an UpSet diagram, there is a vertical bar graph above a table with dots and lines, which is left of a horizontal bar graph. The rows of the table correspond to item types. The columns of the table correspond to particular combinations of items. Vertical lines connecting dots in relevant rows indicate combination of items purchased. The vertical bar graph has a bar for each column in the table to indicate the number of customers who purchased that particular combination of items. The horizontal bar graph has a bar for each row in the table to indicate the total number of customers who purchased each item type irrespective of other purchases.

#### Item combinations (Radial Sets plot) panel

**Fig 9:**
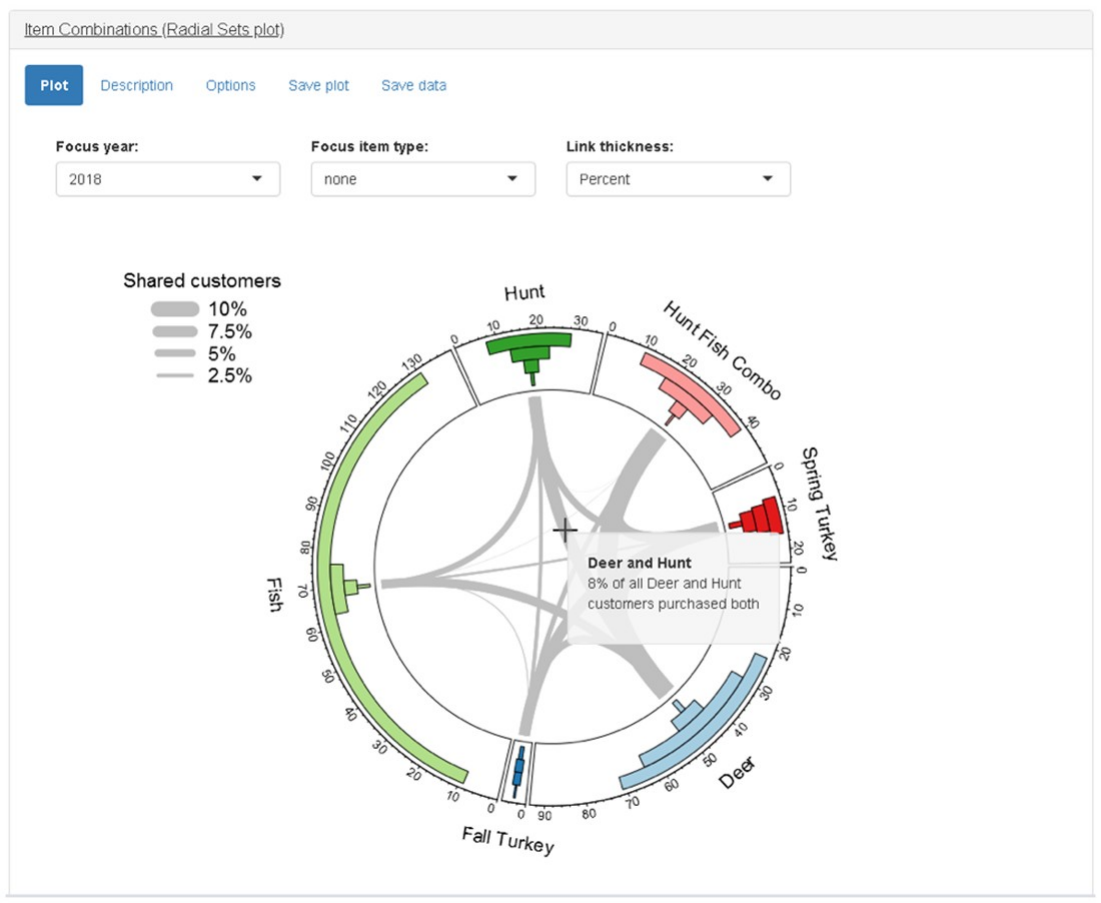
Screenshot of item combinations (Radial Sets plot) panel.

**Fig 10:**
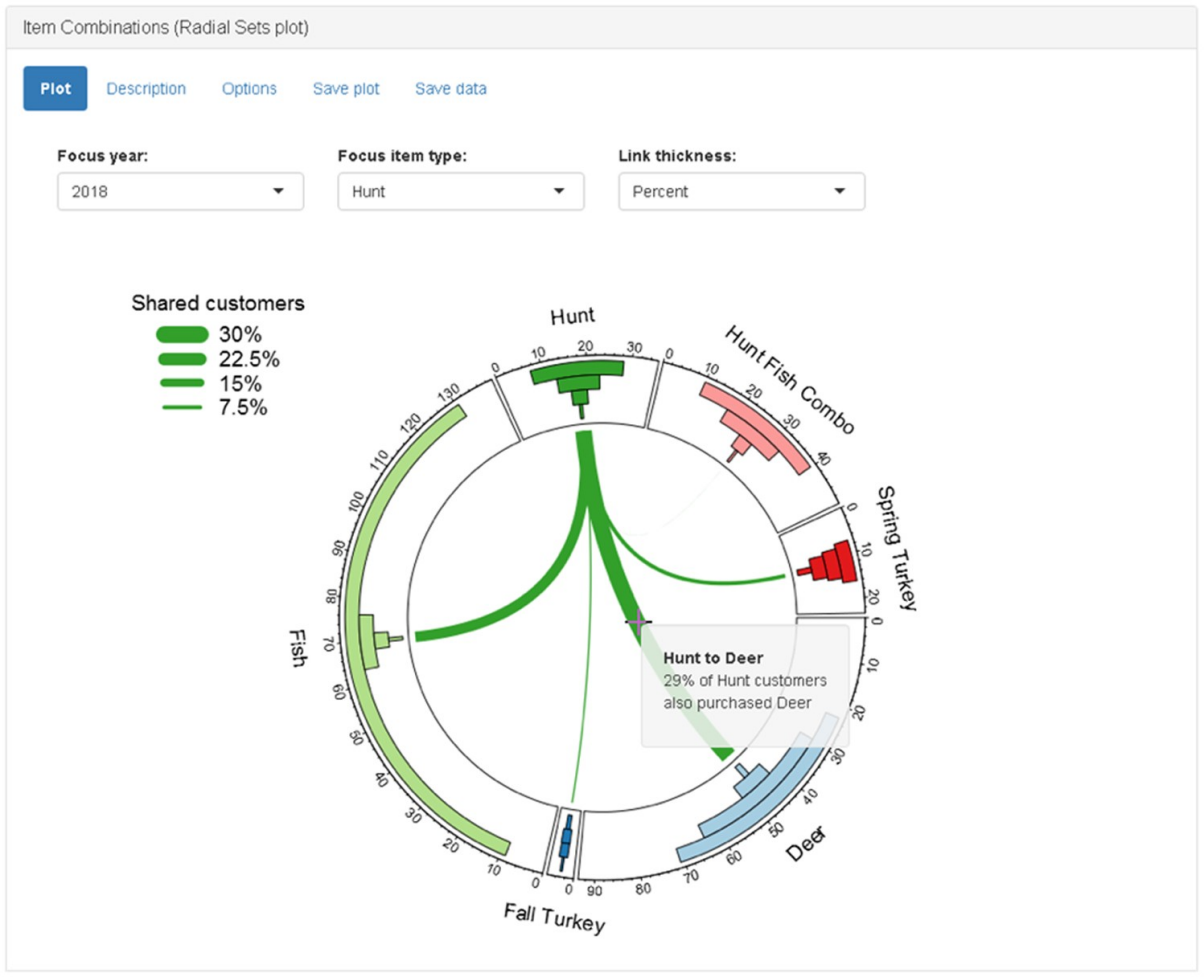
Screenshot of item combinations (Radial Sets plot) panel when “hunt” is selected as focus item type.

Groups of customers purchasing different items form overlapping sets, as explained in the Item Combinations Panel. Network analysis provides another way to visualize these overlapping sets. The permit combination panel displays a graphic based on a Radial Sets diagram (Fig 9) [26]. In our simplified version of a Radial Sets diagram, there is a donut chart with sections for each permit and stamp type. A donut chart is essentially a pie chart with the inside removed to form a ring with sections proportional to the size of each group. The sections are sized according to the total number of customers who purchased each item type irrespective of other purchases. Inside each section of the donut chart, there are four bars indicating the number of customers who purchased only that item type, one additional type, two additional types, or three or more additional types. Inside the ring of the donut plot, there are edges (i.e., lines) connecting the nodes (i.e., sections). The width of the edges indicates the percent overlap between the two customer groups relative to the total number of customers in both groups. For example, the edge between the “hunt” group and the “deer” group indicates the percent of all hunt and deer permit-buyers who purchased both permits. A drop-down menu input allows the user to select between sizing the edges by percent or number of customers. The “focus item type” drop-down menu input allows the user to select a single item type on which to focus. If the user selects an item type from the “focus group” menu, then all edges not connected to that type are removed. The edges are rescaled to indicate the percent of customers in the focus group who also purchased each additional type (Fig 10).

#### Save plot tab

Each panel has a save plot tab. The save plot tab has options to specify a filename and download the plot. The plot tab has options to change the font size, text color, text label angle for the x-axis, background color, plot height, plot width, legend position, and whether the title is included on the plot. A dropdown menu with plot themes is also available to allow the user to select a common combination of plot options. The plot themes include document with light or dark background and presentation with light or dark background.

#### Save data tab

Each panel has a save data tab. The save data tab has options to specify a filename and download a CSV file containing the data displayed in the plot. The tab also displays an interactive table that allows the user to inspect the data before downloading.

## Discussion

We developed the “huntfishapp” R package to meet specific needs of the Nebraska Game and Parks Commission. We have also given consideration in the design to how the app can be used by other state and provincial fish and wildlife agencies and non-governmental organizations. In theory, it should be relatively easy to connect the app to a similar database once a view has been created containing the required data in the specified format. The “huntfishapp” R package is open-sourced, covered under the CCBY 4.0 license, and archived on GitHub. GitHub provides a repository to share improvements, track bugs, and allow users to fork the repository. Researchers or managers with basic R coding skills and interests in developing a similar application can fork the repository and modify the app to meet their own needs or correct bugs, and thus contribute to the ongoing development of “huntfishapp”. We invite readers to provide feedback and make changes to the “huntfishapp” package on GitHub (github.com/chrischizinski/huntfishapp).

Given the complexity and skills required to interact with customer data in electronic licensing systems, it is important to create tools that streamline analyses and provide useful summaries of data to researchers and managers. Tools, like “huntfishapp,” allow users to interact with real-time data on sportspersons without the need to code or directly analyze the data. Web-based interactive decision-support tools provide an opportunity to develop cross platform, highly customizable interfaces, making up-to-date information available for many different applications by state and provincial fish and wildlife agencies and non-governmental organizations. Until recently, access to such tools was limited to individuals with expensive software and web-development expertise; improvements of open-source software grant access to such tools for individuals with inexpensive software and minimal expertise.

While designing the app we tried to balance the need to provide a high-level overview with minimal user input for basic users (i.e., data dashboard style app) with the need to allow more advanced users to dig deeper into the available data by focusing the analysis on specific customer segments (i.e., exploratory data analysis style app). The analysis and graphic functions that are used to construct the app are well documented and available within R when the “huntfishapp” package is installed locally. Users who are comfortable programming in R can use the functions provided by the package to develop custom analyses or in combination with R Markdown to generate reports and presentations. How to best accommodate a variety of users is an important design consideration for organizations developing similar apps.

The “huntfishapp” provides an open-sourced flexible dashboard that can readily be adapted to permit-license or organizational-membership databases. The dashboard can be set up to directly interact with a SQL database or a flat file (e.g., *.csv). The most difficult part in adapting a database would be setting the variable names the same between the users permit-license database and the app; this could readily be accomplished by creating a view in the database or exporting the SQL query to a flat-file. Our dashboard was coded in Shiny, which has at least two drawbacks. First, it can be slower with large queries. We have attempted to address this by allowing a single R process to orchestrate multiple tasks in the background as well as parallel computation. Second, the free version of Shiny Server is limited to a Linux server and limits the number of simultaneous users and will only be a problem if the users wanted a central site to work with the app. The app works well on an individual’s personal computer as long as the computer can interact with the database. In our conversations with NGPC there are a few items that routinely come up. One, there is some training that is needed to understand how to use the app. It is not as simple as a traditional marketplace dashboard that displays plots of sales, customers, and other information in a standardized format. Though this information can be readily gained from the app, we built in greater flexibility for users to explore a lot more of the data than just trend lines. However, this comes at a cost because there is some training is needed. We have written some vignettes and help features into the app, but some work will need to be done by the user to become familiar with the system. Two, users stated that they wish that they could save queries and just be able to click on that stored query. This is a difficult feature to build into the app as it is set up now but could be a nice feature added in a future version. Three, there is concern about interpreting the plots or the data exported from the app. For example, two users may create plots of participation rates but have subtle differences in the queries that could lead to differences in interpretation. To partially address this, we have provided an output of metadata of the queries parameters with the plots or datasets. The NGPC is also discussing how to limit the number of users to a core group in the agency to help moderate and interpret what are being done with the data.

Other organizations seeking to adapt the app for their own needs should also consider the questions they wish to answer, and the type of data-analysis required. A key characteristic of hunting and fishing permit purchase data is that purchases take place in a non-contractual setting. When hunters and anglers have not recently made a purchase, it is not clear if they have permanently quit or are just temporarily not purchasing. The moving window analyses used in the app to estimate customer churn and recruitment are simple model-free methods that can be easily explained to all users. However, model-based methods might be used to provide more accurate estimates if the app was being designed for a more technical audience. Another characteristic of hunting and permit purchase data is a relatively small number of items for sale. When the number of items is relatively small, the bar chart (e.g., UpSet plot) or network-based visualization (e.g., Radial Sets plot) used in the app is suitable for visualizing the overlap of different customer groups. When designing a similar app, or customizing the app, organizations should also consider the full spectrum of available data and how it can be integrated. Future work on “huntfishapp” will seek to combine a variety of data sources including survey responses and event participation.

The decline in the number of people engaging in hunting and fishing activities has placed increased emphasis on efficient deployment of resources. Private businesses were faced with similar circumstances during the downturned economy of the late 2000s [12] and there was an essential need to link marketing resource deployment with performance [27] as well as marketing productivity with performance [10]. The marketing dashboard emerged as a visualization tool for a greater number of users in a company to have access to important metrics on marketing efforts and initiatives [28]. The marketing dashboards helped businesses in two important ways [12]: (1) a dashboard serves as an informational resource and tool that provides a framework to share marketing information across an organization, and (2) a dashboard provides an unbiased metric of performance, which facilitates evaluation and learning [29]. Further, the interactive effects (i.e., different business resources on firm-specific business processes) of marketing dashboards allowed managers across an entire organization to understand and resolve challenges through shared interpretation [12, 30]. Though we fully realize there are fundamental differences in missions between fish and wildlife agencies and for-profit businesses, there are also similarities. Like for-profit businesses, fish and wildlife agencies and non-governmental organizations require understanding of (a) their customers and (b) how management decisions affect customers. Web-based applications, like “huntfishapp,” provide a fully customizable visual representation of sportsperson (customer) metrics that allow for greater evaluation of marketing and R3 efforts.

Fish and wildlife agencies, as well as numerous non-governmental organizations, are increasingly directing resources toward efforts to maintain or increase participation in hunting and fishing. Given the financial challenges facing most conservation organizations, there is an increasing need to evaluate and optimize R3 efforts to produce the greatest effects on sportsperson participation rates. Granting all individuals in an agency with access to information (data) necessary to evaluate marketing and R3 efforts is an important step toward improving the efficiency of efforts to increasing participation in hunting and fishing; an app, like “huntfishapp,” provides a simple tool for granting such access. Although the development of new funding models may ultimately shape the future of natural resource management, data apps and dashboards allow fish and wildlife agencies and non-governmental organizations to become more knowledgeable of their customer base and provide a greater understanding of how management decisions affect participation in hunting and fishing.

The “huntfishapp” provides a simple tool to query and display information from a license-permit or organizational-member database. The app provides important information on customers including the number of them, breakdowns among demographics and locations, churn rates, and cross-buying patterns. The tool is primarily intended to meet the needs of agencies in two ways. One, provide basic information to make data driven decisions of a fish and wildlife agency. Two, to provide tools for those agency members that are specifically working on the recruitment, retention, and reactivation of sportspersons. Our app is different than some of the dashboards that are being used by fish and wildlife agencies. We provide a platform that allows for observation of trend data among customers, but we also provide a tool that allows for greater exploration of the data. Our app is also different because it is open-sourced and can be adapted to agencies information technology systems. There is no requirement of subscription fees or sharing of data to an outside agency. It is our hope that the app is adopted by agencies and continued to be improved and customized to meet the individual needs of state agencies.
